# Pulmonary Manifestations of Sjögren's Syndrome: A Case Report and Review of the Literature

**DOI:** 10.7759/cureus.104601

**Published:** 2026-03-03

**Authors:** Maria Jaquez Duran, Diaz Saez Yordanka, Sindhaghatta Venkatram, Tamera Paczos, Gilda Diaz-fuentes

**Affiliations:** 1 Medicine, BronxCare Health System, Bronx, USA; 2 Pulmonary Medicine, BronxCare Health System, Bronx, USA; 3 Pulmonary/Critical Care, BronxCare Health System, Bronx, USA; 4 Pathology, BronxCare Health System, Bronx, USA

**Keywords:** autoimmune lung disorder, interstitial lung disease with pulmonary hypertension (ild-phtn), nonspecific interstitial lung disease (nsip), sjogren's, ss-ild

## Abstract

Sjögren’s syndrome (SS) is a chronic autoimmune disorder characterized by lymphocytic infiltration of exocrine glands, resulting in xerostomia and keratoconjunctivitis sicca. Beyond glandular involvement, systemic manifestations, particularly pulmonary complications, significantly contribute to morbidity and mortality in affected individuals.

Pulmonary complications in SS range broadly, encompassing interstitial lung conditions, airway inflammation, nodular formations, cystic abnormalities, and pulmonary hypertension. Diagnosis relies on high-resolution computed tomography, pulmonary function tests (PFTs), and, in selected cases, histopathologic evaluation.

We describe the cases of two patients with primary SS presenting with distinct pulmonary findings: one with an incidental pulmonary nodule and the other with organizing pneumonia on a background of cellular non-specific interstitial pneumonia. These presentations illustrate the clinical heterogeneity and diagnostic complexity of pulmonary involvement in SS.

These cases highlight the importance of recognizing SS-related pulmonary complications, which may present subtly and require multidisciplinary evaluation. Increased awareness among healthcare providers and early diagnostic workup are essential to improve patient outcomes.

## Introduction

Sjögren’s syndrome (SS) is a persistent autoimmune condition involving immune cell infiltration and damage to exocrine glands, particularly those responsible for saliva and tear production. Clinically, it manifests as xerostomia (dry mouth) and xerophthalmia (dry eyes) [1,2]. SS is one of the most prevalent systemic autoimmune diseases, second only to rheumatoid arthritis, with an estimated global prevalence of 0.1-0.6% [1]. The condition exhibits a strong female predominance (approximately 9:1), with the majority of cases diagnosed between 40 and 60 years of age [2]. Epidemiological data reveal geographic and ethnic variability, with higher prevalence reported in Northern Europe compared to Asian and African populations [3].

SS may present as either primary Sjögren’s syndrome (pSS) - occurring in isolation - or secondary Sjögren’s syndrome (sSS), which is associated with other autoimmune diseases such as rheumatoid arthritis, systemic lupus erythematosus (SLE), systemic sclerosis, and polymyositis/dermatomyositis [3,4].

From a serological perspective, SS can be further subclassified into seropositive and seronegative forms. Seropositive SS is associated with the presence of autoantibodies including anti-Ro/SSA, anti-La/SSB, rheumatoid factor (RF), anti-citrullinated peptide antibodies (ACPA), and hypergammaglobulinemia [4]. In contrast, seronegative SS lacks these serologic markers but meets the clinical and histopathologic criteria outlined by the ACR/EULAR classification systems [2].

While glandular involvement is the hallmark of SS, extraglandular manifestations are increasingly recognized and may involve the pulmonary, renal, neurologic, hepatic, and vascular systems. Among these, pulmonary involvement is particularly important due to its potential to significantly affect morbidity, quality of life, and prognosis.

In this report, we present two patients with pSS who developed unusual and diagnostically challenging pulmonary manifestations, including a solitary pulmonary nodule and organizing pneumonia. These cases underscore the need for increased clinical vigilance and multidisciplinary collaboration in the evaluation and management of SS-related lung disease.

## Case presentation

Case 1

A 61-year-old African-American woman, never-smoker, with a known history of pSS managed conservatively with artificial tears for xerophthalmia, presented for routine asthma follow-up. Her comorbid conditions included asthma, post-traumatic stress disorder, and depression. Although her asthma was well controlled, she reported pleuritic right-sided chest pain of several weeks' duration. She denied trauma, travel, fever, weight loss, or other systemic or respiratory symptoms.

**Figure 1:**
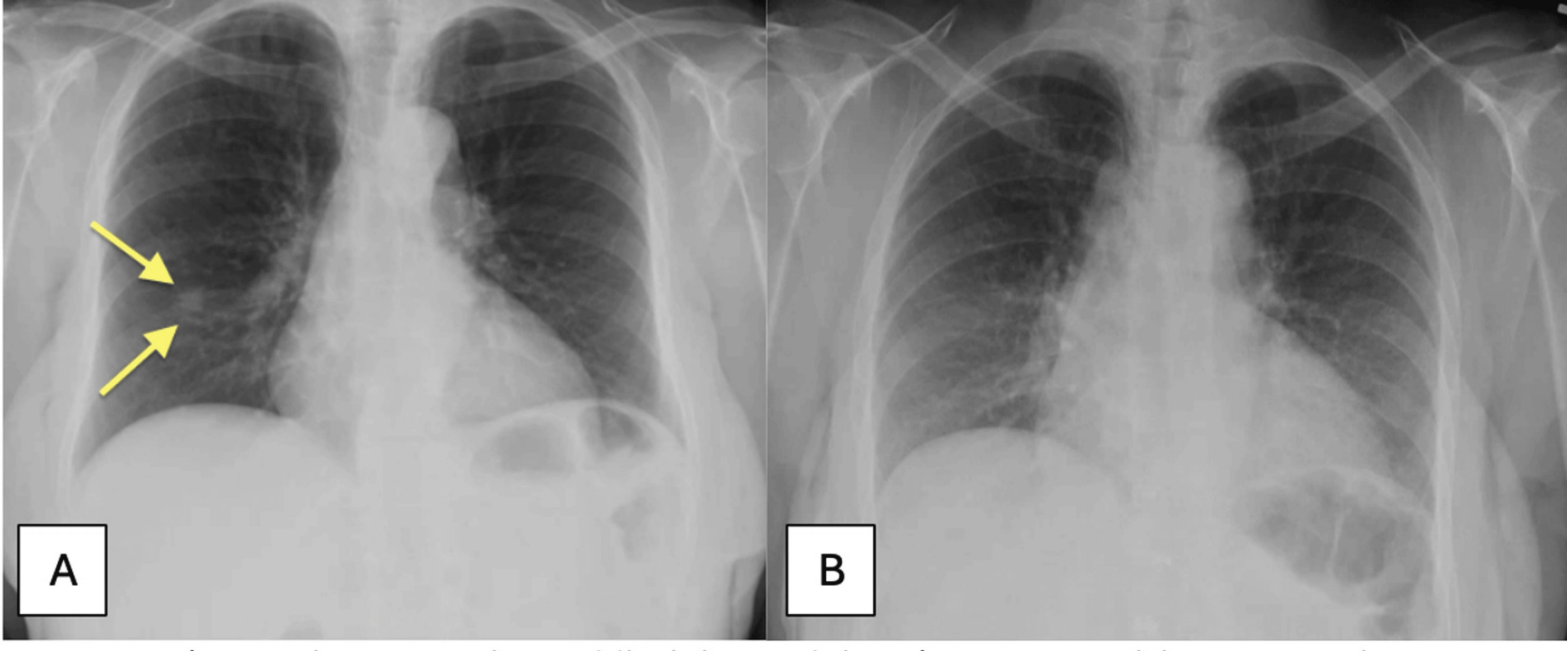
(A) CXR showing a nodule in the right middle lobe. (B) CXR normal lung parenchyma one year prior. CXR, chest X-ray

On physical examination, her vital signs were within normal limits. The only notable finding was localized tenderness over the right anterior chest wall near the third and fourth ribs. No palpable mass was present. A chest radiograph (CXR) revealed a new pulmonary nodule in the right middle lobe (Figure 1A); comparison with a CXR from the previous year showed no abnormalities (Figure 1B).

**Figure 2:**
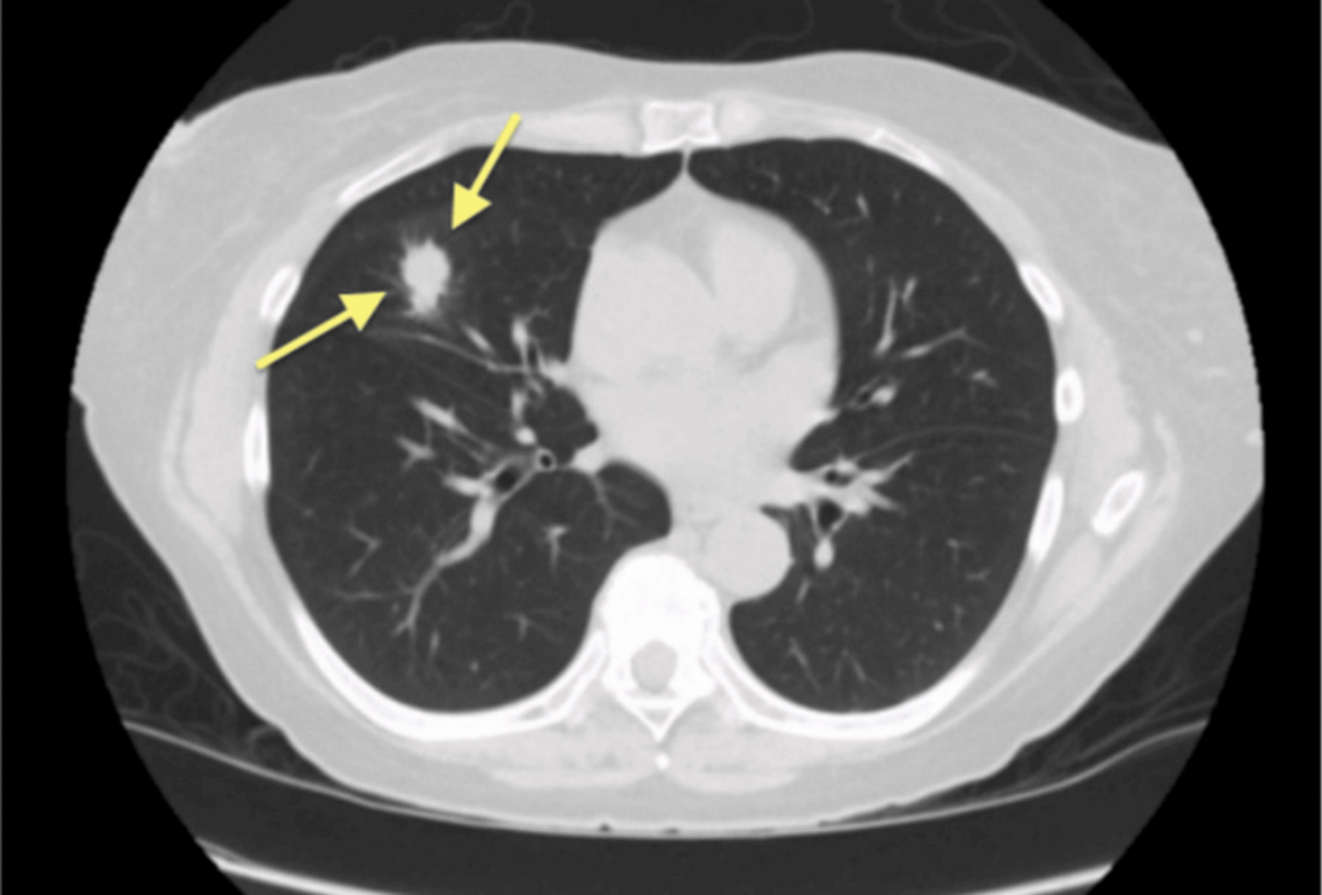
CT of the chest showing a nodule in the right middle lobe. CT, computed tomography

CT imaging of the chest identified a spiculated lesion measuring 1.3 by 1.7 centimeters located in the right middle lobe. (Figure 2). Given the radiographic appearance and clinical context, there was high suspicion for malignancy. A fiberoptic bronchoscopy (FOB) with endobronchial ultrasound-guided transbronchial needle aspiration (EBUS-TBNA) was performed but was non-diagnostic.

**Figure 3:**
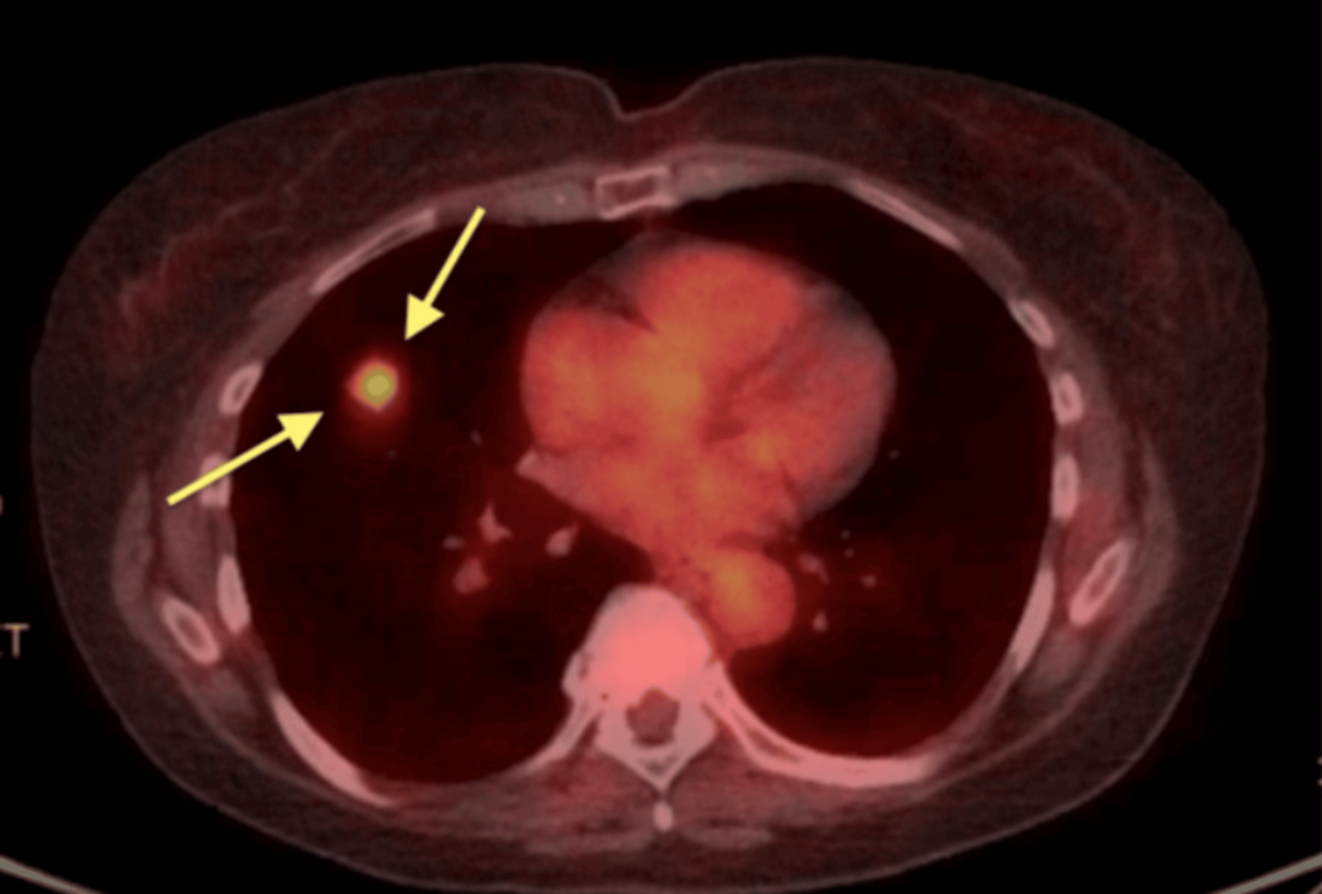
PET scan showing a PET-avid lesion in the right middle lobe (yellow arrow). PET, positron emission tomography

Subsequent positron emission tomography (PET) imaging showed the nodule to be hypermetabolic, with a maximum standardized uptake value (SUV) of 5.0 (Figure 3), further raising concern for malignancy.

**Figure 4:**
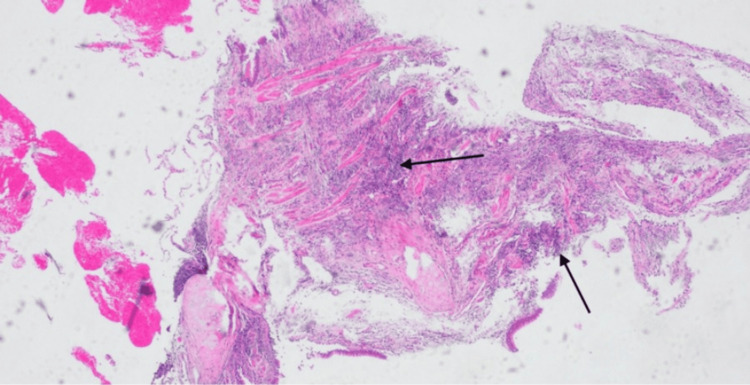
H&E-stained section (magnification 40x) showing lung parenchyma with chronic lymphocytic infiltrates and rare eosinophils, suggestive of bronchiolitis. H&E, hematoxylin and eosin

**Figure 5:**
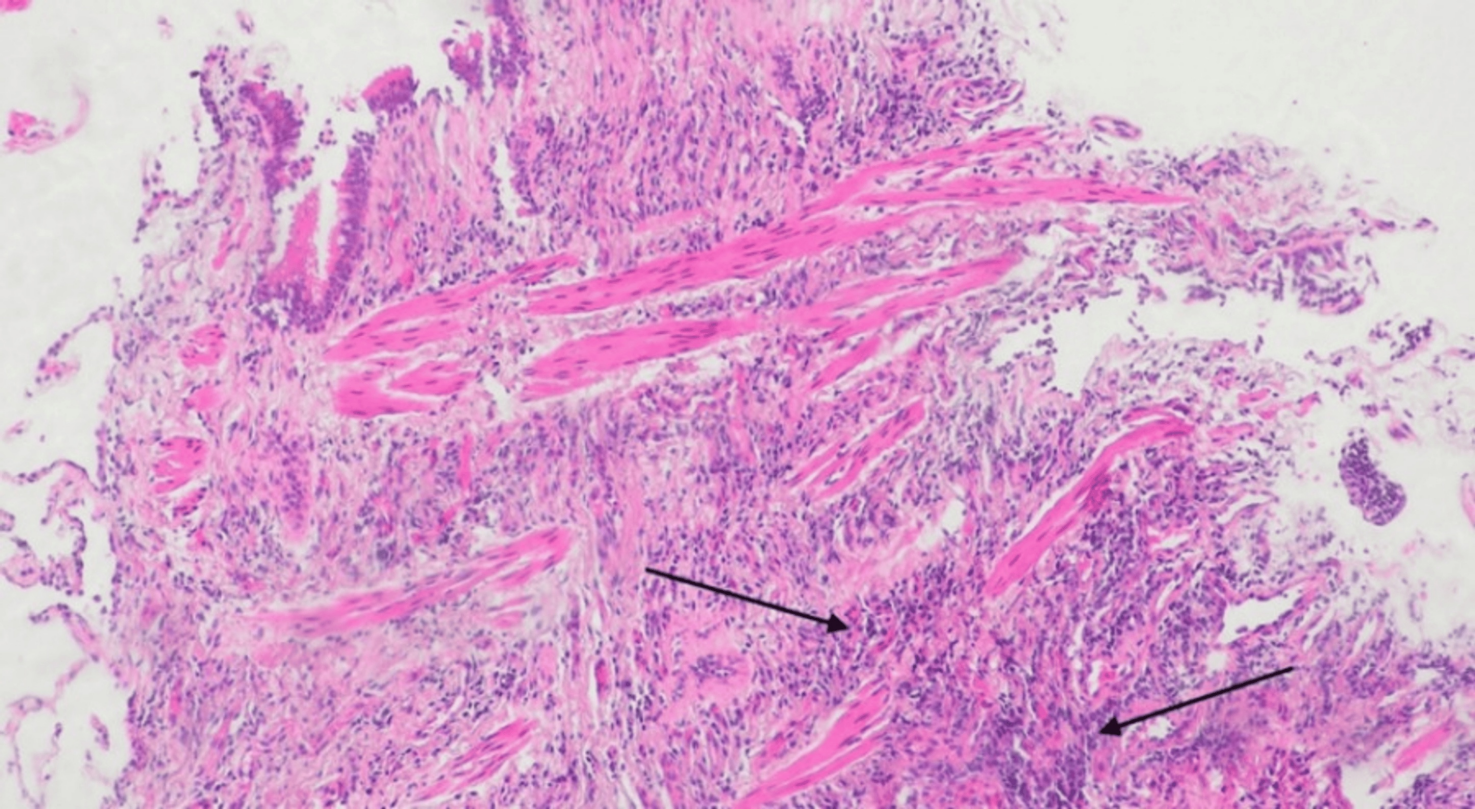
H&E-stained section (magnification 10x) showing lung parenchyma with chronic lymphoplasmacytic infiltrate. H&E, hematoxylin and eosin

The patient underwent video-assisted thoracoscopic surgery (VATS) with right middle lobectomy and regional lymph node dissection. Histopathological evaluation of the nodule revealed chronic lymphocytic infiltrates and rare eosinophils, consistent with bronchiolitis and necrotizing granulomatous inflammation (Figures 4, 5). Tissue cultures for acid-fast bacilli, fungi, and bacteria were negative, and QuantiFERON-TB Gold testing was also negative. Importantly, there was no histologic evidence of malignancy or lymphoma.

The patient was monitored closely over a seven-month follow-up period, during which she remained clinically stable, asymptomatic, and without recurrence of pulmonary findings on serial imaging.

Case 2

A 47-year-old Hispanic female patient, originally from the Dominican Republic and with no smoking history, presented to the ICU due to a sudden onset of shortness of breath, fever, and chills that began the previous day. Her medical background included asthma and high blood pressure. Her symptoms were accompanied by progressive unintentional weight loss of approximately 100 pounds over three years. She reported a prior hospitalization in her home country for a respiratory illness, though no medical records were available for review.

**Figure 6:**
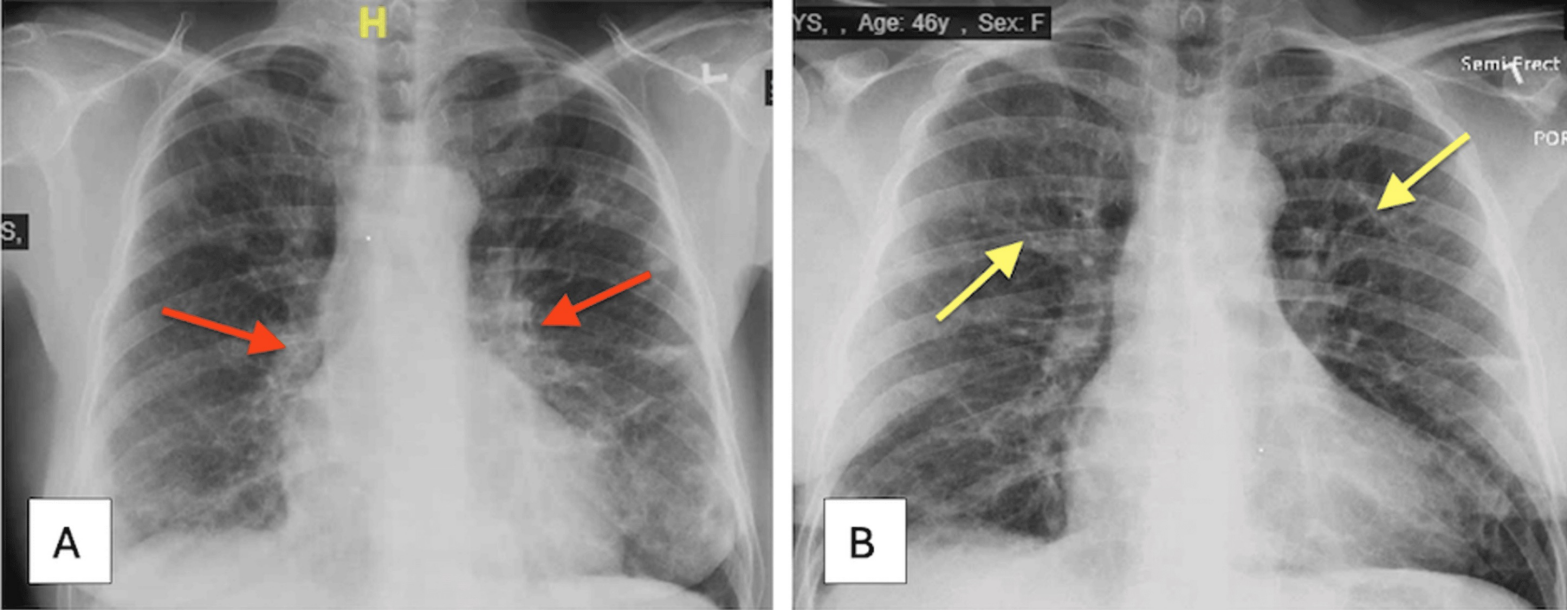
Chest X-ray showing (A) generalized diffuse vascular markings (red arrows) and (B) chronic interstitial lung changes (yellow arrows).

On presentation, the patient was hypoxic, requiring supplemental oxygen. Physical examination was otherwise unremarkable. A CXR on admission demonstrated diffuse vascular markings (Figure 6A), and comparison with a CXR from one month earlier revealed chronic interstitial changes (Figure 6B).

**Figure 7:**
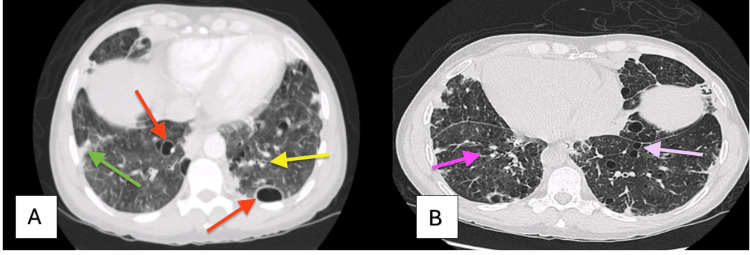
(A) CT of the chest showing diffuse cystic lesions (red arrows), bronchiectasis (yellow arrow), and ground-glass opacities (green arrow). (B) CT of the chest showing bilateral bronchiectasis (pink arrow) and multifocal cystic changes (light pink arrow).

High-resolution chest CT revealed widespread cystic changes, airway dilation consistent with bronchiectasis, and areas of hazy lung opacification (Figure 7), raising suspicion for lymphocytic interstitial pneumonia (LIP) with possible superimposed infection.

Laboratory investigations revealed normocytic anemia, normal white blood cell count, and preserved renal function. Infectious work-up was notable for positive influenza A on viral PCR and *Klebsiella pneumoniae* in urine culture. The patient was treated with oral oseltamivir for influenza, intravenous antibiotics for presumed bacterial pneumonia and urinary tract infection, and oxygen supplementation.

Further serologic evaluation showed strongly positive anti-SSA and anti-SSB antibodies, as well as rheumatoid factor (RF) and anti-citrullinated peptide antibodies (ACPA). N-terminal pro-brain natriuretic peptide (pro-BNP) was elevated at 477 pg/mL, though no clinical heart failure signs were present.

**Figure 8:**
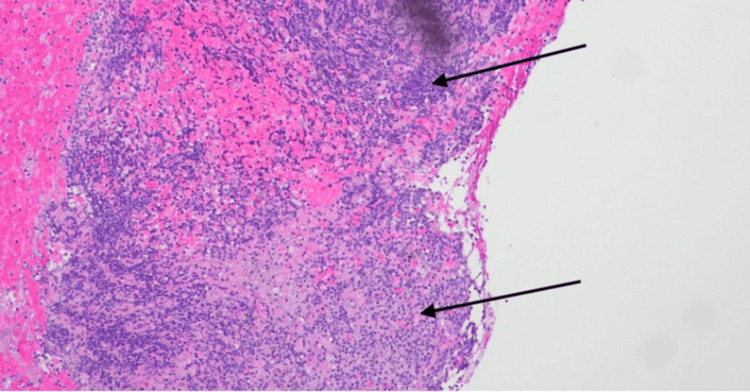
H&E-stained section (magnification 20x) showing lung parenchyma with chronic lymphoplasmacytic infiltrate. H&E, hematoxylin and eosin

Given the radiologic findings and serologic profile, there was high clinical suspicion for pSS-associated cystic lung disease. She underwent FOB with EBUS and transbronchial biopsy, and histopathological examination demonstrated organizing pneumonia on a background of cellular interstitial pneumonitis, suggestive of either a single inflammatory process or a dual pathology (Figure 8). No granulomas, infectious organisms, or aspirated material were identified. The differential diagnosis included collagen vascular disease, infection, aspiration, and drug toxicity.

After consultation with rheumatology, a working diagnosis of pSS-associated cystic lung disease with organizing pneumonia was made. She was initiated on methylprednisolone 62.5 mg daily, with marked symptomatic improvement, and was later discharged on a tapering dose of prednisone.

**Figure 9:**
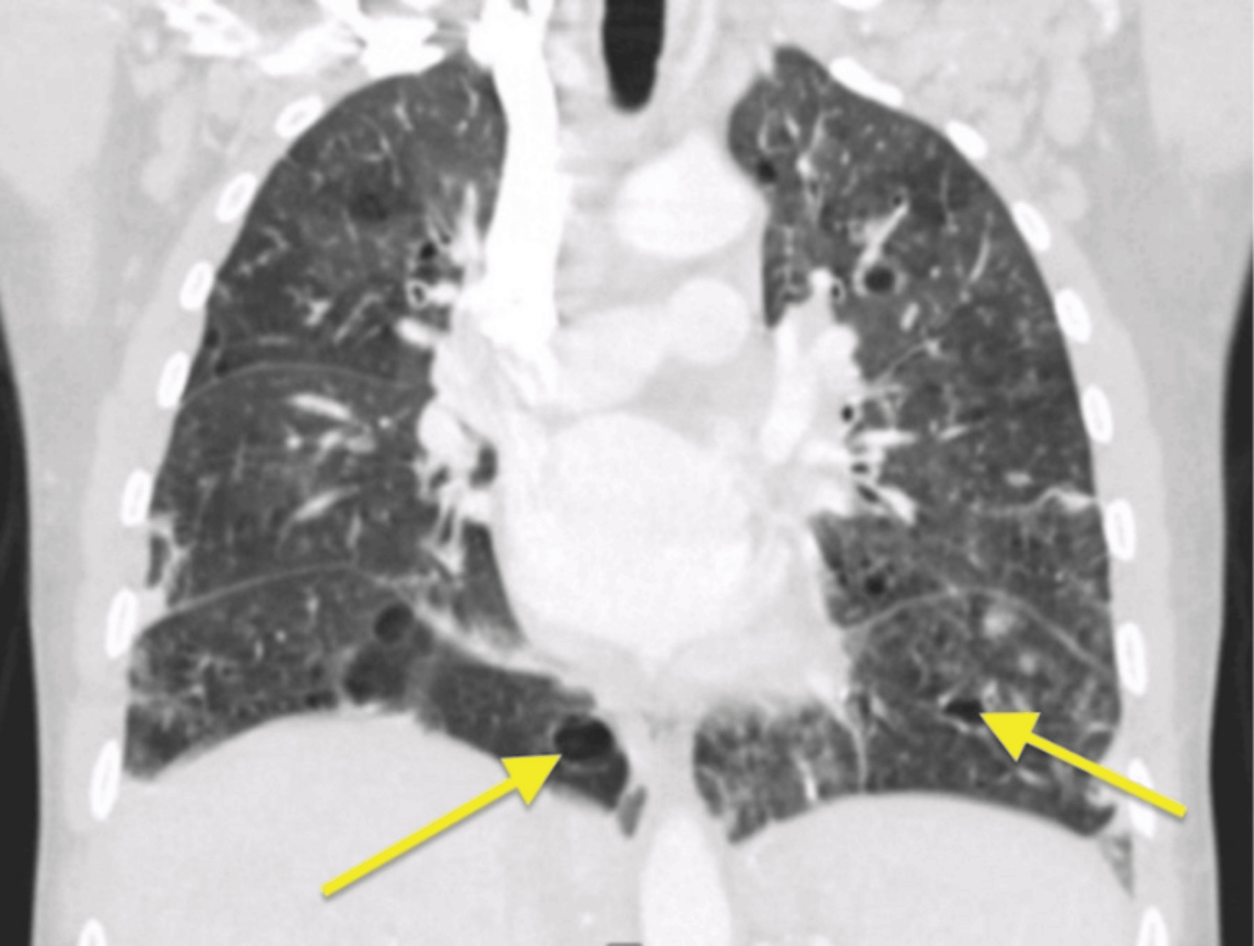
CT scan of the chest showing diffuse cystic lesions (yellow arrows).

On outpatient follow-up, the patient remained clinically stable, with resolution of respiratory symptoms and stabilization of body weight. Pulmonary function testing (PFT) showed a mixed obstructive and restrictive pattern, with an FEV₁ of 2.09 L (44% predicted) and a severely reduced diffusing capacity for carbon monoxide (DLCO) (39% predicted). Repeat CT of the chest performed one month later demonstrated persistent bilateral bronchiectasis and multifocal cystic lung changes (Figure 9).

She continued therapy with prednisone 40 mg daily for five months, which was then tapered. Due to ongoing pulmonary involvement, she was started on mycophenolate mofetil for immunosuppressive maintenance. She remains under close follow-up with both rheumatology and pulmonary specialists.

## Discussion

The underlying mechanisms of pSS are multifactorial, involving genetic risk factors, environmental stimuli, immune imbalance, and hormonal contributions. [2]. Genetic susceptibility includes specific HLA haplotypes (e.g., HLA-DRB1, HLA-DQB1) and polymorphisms in genes such as STAT4, IRF5, and BLK [1,3]. Environmental factors, particularly viral infections such as Epstein-Barr virus, cytomegalovirus, hepatitis C, and HTLV-1, are proposed triggers. Hormonal influences, including estrogen deficiency, may help explain the higher prevalence of pSS among postmenopausal women [1,2].

In the lungs, chronic immune activation causes inflammatory injury, lymphocytic infiltration, and progressive fibrosis. These mechanisms underlie interstitial lung disease (ILD) patterns such as LIP and non-specific interstitial pneumonia (NSIP), as well as small airway disease, including bronchiolitis [5]. Persistent inflammation can also predispose to lymphoproliferative complications, such as mucosa-associated lymphoid tissue (MALT) lymphoma [4].

Clinical respiratory symptoms in pSS are often non-specific and may include dyspnea (62%) and cough (54%). Other symptoms include sputum production, chest pain, and fever. Despite the breadth of potential pulmonary involvement, manifestations are frequently asymptomatic and detected incidentally, with reported prevalence ranging from 8% to 80%, depending on the diagnostic modality. Approximately 20% of pulmonary abnormalities are considered clinically significant. The cumulative incidence of ILD in pSS is estimated at 10-20% within one and five years of diagnosis, respectively [6].

ILD as the presenting manifestation of pSS, as observed in our second patient, is rare [6]. The clinical features of SS vary by organ involvement and can be categorized into glandular and extraglandular manifestations. While glandular symptoms dominate the clinical picture, extraglandular involvement, including pulmonary, renal, hepatic, neurological, and vascular systems, is not uncommon [1]. Disease severity can range from isolated sicca symptoms to severe systemic involvement, including ILD, vasculitis, or lymphoma [3].

Our first patient was found to have an incidental pulmonary nodule. Although the prevalence of nodules in pSS is not well established, their occurrence is thought to be related to B-cell lymphoproliferative activity and increased immunoglobulin deposition in lung tissue. Nodules can be solitary or multiple. In MALT lymphoma, nodules often present with associated ground-glass opacities or airspace consolidation, typically distributed along bronchovascular bundles and interlobular septa. A Mayo Clinic case series of patients with pSS and pulmonary nodules who underwent biopsy found the following diagnoses: non-Hodgkin lymphoma (39%), MALToma without amyloid (22%), MALToma with amyloid (15%), diffuse large B-cell lymphoma (2%), lung carcinoma (27%), granulomas (7%), focal organizing pneumonia (5%), and benign disease (29%) [6].

Granulomatous inflammation is relatively rare in pSS and presents a diagnostic challenge. It may mimic infectious or other autoimmune granulomatous diseases. Rarely, conditions such as Kikuchi-Fujimoto disease (histiocytic necrotizing lymphadenitis) can be seen, particularly in young women [5]. Fortunately, our patient’s granulomatous inflammation was benign and attributable to pSS, suggesting a favorable prognosis.

Our second patient had a more complex presentation with cystic changes, ground-glass opacities, and bronchiectasis on chest CT. PFT demonstrated both obstructive and restrictive physiology with diffusion impairment. Lung biopsy revealed organizing pneumonia superimposed on a background of cellular NSIP. Among pSS-associated ILD patterns, NSIP is most common, followed by usual interstitial pneumonia (UIP) and LIP. Organizing pneumonia, particularly cryptogenic organizing pneumonia, is rarely seen in pSS. In our case, it may have been related to a preceding viral infection, as has been reported in influenza-related organizing pneumonia [7,8]. Bronchiectasis occurs in up to 10% of patients with pSS and can predispose to recurrent infections and obstructive lung disease.

ILD in pSS is frequently associated with positive anti-SSA antibodies, low complement levels (particularly C3), and elevated inflammatory markers such as rheumatoid factor and C-reactive protein. Compared to idiopathic pulmonary fibrosis with a UIP pattern, pSS-related UIP tends to affect older women, shows more pronounced bronchial thickening on imaging, and generally carries a better prognosis with responsiveness to immunosuppressive therapy. In contrast, LIP has a variable course, ranging from resolution to progression into lymphoma and death [6].

Cystic lung disease, typically bilateral and affecting mid-lung zones, as seen in our second patient, is generally considered benign and not linked to increased risk of malignancy. The development of cysts has been associated with anti-SSB antibody positivity [6].

Our patient’s PFT findings were consistent with small airway disease, a common feature in symptomatic pSS. This includes bronchiectasis (often anti-smooth muscle antibody positive and predominantly affecting the lower lobes), bronchiolitis, and reactive airway disease. In cases with concomitant parenchymal involvement, a restrictive pattern with reduced diffusion capacity may also be observed.

Additional pulmonary manifestations of pSS include amyloidosis, vascular disease, lymphatic involvement, and pleural effusions. Pulmonary amyloidosis, typically of the AL subtype, may involve the interstitium or airways and presents with symptoms such as cough, dyspnea, hemoptysis, and pleuritic pain. Amyloidosis can occur in isolation or secondary to lymphoproliferative disease. Diagnosis is confirmed with Congo red staining, which reveals birefringence under polarized light. Biopsy may be required to exclude lymphoma [4,8,9].

The chronic inflammatory environment in pSS fosters the development of extranodal marginal zone B-cell lymphomas, particularly MALT lymphoma. Histologically, these lesions show monoclonal B-cell proliferation, often with plasmacytoid features that may resemble benign infiltrates. Primary pulmonary lymphomas, including bronchial-associated lymphoid tissue (BALT) lymphoma, are found in approximately 2% of pSS patients and are thought to result from chronic antigenic stimulation [4].

Pulmonary hypertension is a rare but serious complication of pSS, with a reported one-year survival of 70% and three-year survival of 65%. Its pathogenesis is multifactorial and includes ILD, vasculopathy (e.g., arteriopathy, veno-occlusive disease), and valvular heart disease. Treatment is not well defined. Immunosuppressive therapy may provide some benefit, and in certain cases, second-line pulmonary vasodilators are considered. Importantly, pSS-associated pulmonary hypertension patients often do not respond to vasodilator challenge, and calcium channel blockers are generally ineffective [9].

Patients with pSS are also at an increased risk for pulmonary embolism, likely due to both a pro-inflammatory state and the presence of antiphospholipid antibodies (e.g., anticardiolipin, anti-β2GP1, lupus anticoagulant). Despite these associations, the exact prevalence of venous thromboembolism in pSS remains uncertain [9].

Pleural involvement in pSS is rare and more frequently reported in patients from Japan and Europe. It is often associated with overlapping conditions such as rheumatoid arthritis, SLE, or lymphoma. Pleural effusions, when present, are typically bilateral, lymphocyte-predominant, and exudative. Pleural fluid analysis may show elevated RF, anti-SSA/Ro, and anti-SSB/La antibodies, and low complement levels may suggest coexisting SLE [9].

Histopathologic and diagnostic evaluation of pulmonary involvement in pSS

Histopathological evaluation is critical for both the diagnosis and classification of pulmonary involvement in pSS. Distinct ILD patterns have been characterized in pSS, each with unique clinical and prognostic implications:

NSIP: it typically presents with consistent inflammation and fibrotic changes in the lung interstitium. The cellular variant is notable for dense lymphoplasmacytic cell presence [9].

UIP: less frequently observed in pSS, UIP portends a poorer prognosis. It is characterized by spatially heterogeneous fibrosis, fibroblastic foci, and honeycombing [9].

LIP: it is marked by widespread infiltration of the alveolar walls with a mix of lymphocytes and plasma cells, typically polyclonal in nature [9].

Organizing pneumonia: identified histologically by the presence of fibromyxoid plugs (Masson bodies) within the alveolar ducts and alveoli, often in a subpleural and peribronchovascular distribution [9].

Airway involvement may manifest as lymphocytic bronchiolitis, which is characterized by peribronchiolar lymphocytic aggregates and germinal center formation [9]. Additionally, cystic lung alterations may be seen in association with LIP, amyloidosis, or pulmonary lymphoma [10].

The diagnosis of pSS is established using either the 2016 ACR/EULAR classification criteria [2] or the 2002 American-European Consensus Group (AECG) criteria [1]. Diagnosis of pulmonary complications relies on a combination of imaging, PFT, and histopathological confirmation when indicated.

High-resolution computed tomography (HRCT) is the imaging modality of choice for evaluating pulmonary involvement [11]. Common findings include airway disease, such as tree-in-bud opacities, air trapping, and bronchial wall thickening [12], and ILD patterns, such as ground-glass opacities, reticulations, cystic changes, and honeycombing [12,13]. Emerging technologies in quantitative imaging and AI-driven image analysis are improving the sensitivity and specificity of ILD diagnosis and monitoring.

Pulmonary function tests (PFTs) in pSS often reveal obstructive physiology in small airway disease, restrictive physiology in parenchymal ILD, and reduced diffusing capacity (DLCO) in NSIP and LIP, making it a sensitive marker for disease progression [9]. Additionally, measurements of total lung capacity and residual volume help distinguish between purely interstitial versus mixed obstructive-restrictive ventilatory defects. A mixed pattern, such as observed in our second case, suggests simultaneous involvement of both airways and lung parenchyma. FOB with bronchoalveolar lavage may reveal a T-cell-predominant lymphocytic alveolitis, though these findings are non-specific. FOB is most useful in excluding infection or malignancy in patients with new or progressive lung findings [9]. When radiologic or clinical features are ambiguous, histopathological confirmation - via transbronchial or surgical lung biopsy - is essential for accurate classification of ILD subtype and exclusion of lymphoproliferative disease.

Management and prognosis

Management of pulmonary complications in pSS is tailored to the specific type of manifestation, as therapeutic responses and prognoses vary widely across phenotypes.

For patients with bronchiectasis, treatment is generally supportive and includes airway clearance strategies such as hypertonic saline nebulization, along with bronchodilators. Chronic macrolide therapy may be considered in those with recurrent infections, though supporting evidence in pSS-specific populations is limited. In contrast, hyperreactive airway disease, which may mimic asthma, often does not respond to anti-inflammatory treatments such as inhaled corticosteroids [8].

In cases of ILD, treatment primarily involves immunosuppressive medications. Drugs such as azathioprine, mycophenolate mofetil, and cyclophosphamide have shown some benefit in limited studies; however, these trials have included few patients with pSS, limiting generalizability. Rituximab, an anti-CD20 monoclonal antibody, has shown promising results in a small open-label trial, with reported improvements in DLCO, dyspnea, fatigue, cough, and CT imaging findings among pSS-ILD patients [8].

Current therapeutic strategies can be grouped into three main categories: (1) immunosuppressive and anti-inflammatory therapy to mitigate autoimmune-mediated parenchymal injury; (2) antifibrotic agents (e.g., nintedanib or pirfenidone) in select patients with progressive fibrosing ILD, though data in pSS remain limited; and (3) supportive measures, including bronchodilators, pulmonary rehabilitation, and supplemental oxygen where indicated.

Despite expanding treatment options, outcomes in pSS-associated ILD remain heterogeneous and are poorly described due to reliance on small cohort studies and case series. In available reports, complete resolution has been observed in up to 14% of patients, clinical and radiologic improvement in up to 57%, and disease worsening in approximately 28% [14,15].

In a large Spanish cohort of 1,580 pSS patients, 208 exhibited severe systemic disease, of whom 25 had pulmonary involvement [16]. Independent risk factors for mortality among pSS-ILD patients included smoking history, reduced DLCO, and decreased maximum expiratory flow at 25% of vital capacity (MEF25%).

Prognosis is influenced by the pattern of lung involvement, age at onset, and rate of progression. While some patients experience slowly progressive or stable disease, others may deteriorate rapidly. The 10-year survival rate among patients with pSS and systemic involvement has been estimated at ~50%, although the five-year survival for pSS-ILD specifically is more favorable, at approximately 84% [17].

Our two cases underscore the diagnostic and therapeutic complexity of pulmonary involvement in pSS. Early recognition and tailored treatment are essential, as even subclinical manifestations may herald progressive or systemic disease.

## Conclusions

Pulmonary involvement in pSS is clinically diverse, encompassing ILD, airway pathology, and lymphoproliferative disorders. These complications can significantly impair quality of life and are associated with increased morbidity and, in some cases, mortality. Notably, pulmonary manifestations may be subtle or asymptomatic, with many detected incidentally during routine imaging or evaluation for unrelated symptoms.

Given the broad spectrum of pulmonary presentations and the often nonspecific nature of symptoms, a high index of clinical suspicion is essential. Regular monitoring with pulmonary function tests and high-resolution chest imaging is recommended to enable early detection and guide timely intervention.

Optimal management strategies remain incompletely defined due to limited high-quality data specific to pSS. As such, there is a pressing need for prospective studies and evidence-based guidelines that address the full range of pulmonary complications in this population. Future research should aim to clarify the natural history, identify prognostic markers, and assess the efficacy of targeted therapies to improve outcomes in patients with pSS-related lung disease.
